# Dark Chocolate Intake May Reduce Fatigue and Mediate Cognitive Function and Gray Matter Volume in Healthy Middle-Aged Adults

**DOI:** 10.1155/2022/6021811

**Published:** 2022-12-13

**Authors:** Kiyotaka Nemoto, Keisuke Kokubun, Yousuke Ogata, Yasuharu Koike, Tetsuaki Arai, Yoshinori Yamakawa

**Affiliations:** ^1^Department of Psychiatry, Faculty of Medicine, University of Tsukuba, Tsukuba, Japan; ^2^Open Innovation Institute, Kyoto University, Kyoto, Japan; ^3^Department of Global Studies, Kyoto Seika University, Kyoto, Japan; ^4^Institute of Innovative Research, Tokyo Institute of Technology, Meguro, Tokyo, Japan; ^5^ImPACT Program of Council for Science, Technology and Innovation (Cabinet Office, Government of Japan), Chiyoda, Tokyo, Japan; ^6^Office for Academic and Industrial Innovation, Kobe University, Kobe, Japan

## Abstract

**Background:**

Dark chocolate has attracted attention for its potential for cognitive improvement. Though some reports indicate that dark chocolate is good for cognitive function, others raise doubts. This inconsistency in past results reflecting the relationship between dark chocolate and cognitive function indicates the potential existence of factors that mediate between dark chocolate intake and cognitive function.

**Methods:**

With the hypothesis that fatigue may be one such mediating factor, we performed a four-week randomized control study to seek a link between dark chocolate consumption, cognitive function, fatigue, and the brain in middle-aged adults.

**Results:**

We found that dark chocolate reduced mental and physical fatigue, and a path analysis revealed that it enhanced vitality, executive function, memory, and gray matter volume both directly and indirectly. Fatigue reduction was also associated with an improvement in physical function, which had a positive impact on emotional functioning, relief of bodily pain, and social functioning.

**Conclusions:**

Our results suggest that dark chocolate may help reduce fatigue in individuals, leading to improvements in brain health and various cognitive functions as well as in quality of life.

## 1. Introduction

Dark chocolate, or cocoa, has attracted attention for its potential for cognitive improvement. Socci et al. reviewed research investigating the relations between cocoa and cognition and suggested that cocoa might lead to dose-dependent improvements in general cognition, attention, processing speed, and working memory [1]. Quite a few studies have reported the positive impact of dark chocolate or cocoa on various domains of cognition. Flavanol-rich chocolate was reported to counteract vascular impairment and restore working memory performance [2]. A randomized controlled trial reported that the short-term consumption of a cocoa beverage augmented the blood oxygenation level-dependent signal intensity response during a cognitive switching task [3]. These positive results may be induced by cocoa flavanols, which have been shown to reduce blood pressure [4], improve endothelial function [5], and increase cerebral blood flow [3].

In contrast to these findings, some researchers have pointed out the weak evidence for the efficacy of dark chocolate or cocoa on positive health outcomes. Veronese et al. performed an umbrella review of systematic reviews of health outcomes associated with chocolate consumption [6]. They reported that though chocolate consumption is associated with reduced risk of diabetes or cardiovascular-disease-related death, the evidence was weak. Regarding cognition, a systematic review showed that chocolate consumption was not associated with better cognitive function [7].

This inconsistency in past results reflecting the relationship between dark chocolate and cognitive function indicates the potential existence of factors that mediate between dark chocolate intake and cognitive function. One possible factor is fatigue. Using a highly demanding cognitive test battery, Scholey et al. showed that mental fatigue was attenuated significantly by the acute administration of cocoa flavanols. Reductions in mental fatigue were also accompanied by improvements in serial subtraction accuracy [8]. Another study comparing polyphenol-rich chocolate with simulated isocalorific chocolate suggests that polyphenol-rich chocolate may improve symptoms in subjects with chronic fatigue syndrome [9]. These results led us to consider that chocolate consumption might reduce fatigue, which has an effect on cognitive improvement. In terms of fatigue, we have revealed that it is associated with gray matter volume [10]. In order to clarify the hypothesis that dark chocolate consumption may reduce fatigue, resulting in the improvement of cognitive function and brain structure, we performed a randomized control study.

## 2. Materials and Methods

### 2.1. Subjects

One hundred and four participants (fifty-two females) were recruited through Internet advertisement. We recruited middle-aged adults ranging from 40 to 65 years old since previous literature reviews indicate that aging might be related to fatigability [11, 12]. Potential participants who had medical histories of diabetes mellitus, neurological conditions, psychiatric conditions, or medical conditions that could affect the central nervous system were excluded from recruitment. Upon recruitment, all participants completed a self-report questionnaire about their daily habits such as smoking, drinking, or sleeping time. This study was approved by the ethics committees of the Tokyo Institute of Technology (A19075) and the University of Tsukuba (No. 1568) and was performed in accordance with the guidelines and regulations of the Tokyo Institute of Technology. All participants gave written informed consent prior to participation, and participant anonymity has been preserved.

### 2.2. Experimental Design and Procedures

This study used a four-week, randomized, fixed-dose, parallel-group experimental design (Protocol number UMIN000038407). The participants were randomly assigned to a dark chocolate intervention group (*n* = 56, 28 females and 28 males, mean age: 52.5 ± 7.2 years) or a control group (*n* = 48, 24 females and 24 males, mean age: 52.6 ± 6.4 years). The study used 72% dark chocolate. Participants assigned to the intervention group were instructed to eat five pieces of dark chocolate, which is equal to consuming 635 mg of cocoa polyphenol, per day for 28 days. Participants were asked to record how many pieces of dark chocolate they ate each day on a recording sheet in order to confirm and measure their consumption. Participants assigned to the control group were instructed not to change their daily lives and not to eat chocolate during the experiment. All participants underwent a follow-up MRI, 28 days after their baseline MRI.

### 2.3. Assessment of Fatigue and Cognitive Function

We used the same questionnaires and cognitive test battery on the first day and the last day of the intervention period. We employed two sets of questionnaires: the Chalder Fatigue Scale (CFS) [13] and the 36-item Short Form Health Survey (SF-36) [14], to evaluate the physical and mental changes in participants. The CFS, one of the most frequently used scales to measure the severity of fatigue, was originally a fourteen-item scale measured on a four-point scale from 0 (less than usual) to 4 (much more than usual). However, an 11-item version, which excludes three items, has also been used in many studies. Both the 14-item version and the 11-item version are divided into two subscales, physical fatigue and mental fatigue. We used the most comprehensive measure, the 14-item version, but will also refer to the results of the 11-item version.

The SF-36 consists of 36 questions which evaluate the health status of participants in eight domains: (1) physical functioning, (2) role-physical (role limitations due to physical functioning), (3) bodily pain, (4) general health, (5) vitality, (6) social functioning, (7) role-emotional (role limitations due to emotional functioning), and (8) mental health. The score for each domain ranges from 0 to 100, with a higher score indicating a better state of health. Using the national norm score and its SD, the scores for each of our participants were transformed to norm-based scores (NBSs). We use the NBSs to compare results between studies [15]. As is shown in Table 1, of all the NBSs for the SF-36, the highest was 56.541 and the lowest was 50.414, indicating that there was no significant difference between groups, and that the difference is within one standard deviation from the national average. Therefore, with regard to the SF-36, the sample reflects the national average.

As for cognitive function, we employed the Trail-Making test (TMT), Stroop test, and Japanese version of the MCI screen (The Medical Care Corporation, Irvine, CA, United States). The TMT is a tool used to measure the cognitive domains of processing speed, sequencing, mental flexibility, and visual motor skills [16]. It comprises parts A and B. In part A, the subject connects a series of 25 numbers in numerical order; and in part B, the subject connects 25 numbers and letters in numerical and alphabetical order, alternating between the numbers and letters. We measured the total time to completion for both parts A and B. Then, we calculated the difference (Part B – Part A), which we call the “TMT score” in this manuscript. The TMT score should reflect cognitive flexibility.

As for the Stroop test, we employed the Japanese version of the Stroop Color-Word Test-Victoria version [17]. We performed this test in the same way as Bayard et al. [18]. The stimuli were presented on three different cards, one for each condition. Four colors, blue, green, yellow, and red, were used. First, color dots were presented (dot condition). Next, the words blue, green, yellow, and red were written in a random order in one of the four colors (word condition). Then, the words blue, green, yellow, and red were written in one of the three other colors (interference condition). Twenty-four stimuli were presented on each of the three cards. Participants were asked to name the color of the dots and the color of the written words as quickly as possible. In the Interference condition, participants had to inhibit the written word in order to correctly name the color of the ink. For each condition, we measured the completion time. Then, the Stroop effect was computed as Interference–Word for time, which we call the “Stroop score” in this manuscript.

The MCI screen is a ten-minute staff-administered test to assess memory, executive function, and language, which was developed based on the protocol of the Consortium to Establish a Registry for Alzheimer's disease 10-word recall test [19]. The Memory Performance Index (MPI) score calculated from the MCI screen has been shown to be effective in detecting amnestic mild cognitive impairment [20]. MPI ranges from 0 to 100 and larger values indicate better performance.

### 2.4. MRI Data Acquisition

All magnetic resonance imaging (MRI) data were collected on the same days we assessed the cognitive function of the participants using a 3-T Siemens scanner (MAGNETOM Prisma, Siemens, Munich, Germany) with a 32-channel head array coil. We used the magnetization-prepared rapid-acquisition gradient echo (MPRAGE) pulse sequence for three-dimensional T1-weighted images with the following parameters: repetition time (TR), 1900 ms; echo time (TE), 2.53 ms; inversion time (TI), 900 ms; flip angle, 9°; matrix size, 256 × 256; field of view (FOV), 256 mm; slice thickness, 1.2 mm. Diffusion data were collected with spin-echo echo-planar imaging (SE-EPI) with GRAPPA (generalized autocalibrating partially parallel acquisitions) with the following parameters: TR: 9,000 ms; TE: 81 ms; flip angle: 90°; matrix size: 114 × 114; FOV: 224 mm; slice thickness: 2 mm. A baseline image (*b* = 0 s/mm2) and 30 different diffusion orientations with a *b* value of 1000 s/mm^2^ were acquired with slices parallel to the orbitomeatal line.

### 2.5. MRI-Derived Brain Measures: GM-BHQ and FA-BHQ

A detailed description of the method used for acquiring GM-BHQ and FA-BHQ is provided elsewhere [21, 22]. Briefly, gray matter images were extracted from T1-weighted images using Statistical Parametric Mapping 12 (SPM12; Wellcome Trust Centre for Neuroimaging, London, UK) running on MATLAB R2018b (Mathworks Inc., Sherborn, MA, USA) on Lin4Neuro [23]. The segmented GM images were spatially normalized using the diffeomorphic anatomical registration through exponentiated lie algebra (DARTEL) algorithm [24] with modulation to preserve GM volume. Normalized GM images were smoothed with an 8 mm full width at half-maximum (FWHM) Gaussian kernel. Proportional GM images were generated by dividing smoothed GM images by the intracranial volume to adjust for differences in whole-brain volume across participants. Then, we calculated the GM brain healthcare quotient (BHQ) using the following formula: 100 + 15 × (individual proportional GM − mean)/standard deviation (SD). Regional quotients were then extracted using an automated anatomical labeling (AAL) atlas [25] and averaged across regions to produce participant-specific GM-BHQs.

Diffusion data were preprocessed using the FMRIB Software Library (FSL) 6.0.4 [26]. First, eddy current distortion correction was performed using eddy_correct, followed by generation of FA images using dtifit. FA images were then spatially normalized using FLIRT and FNIRT. Normalized FA images were smoothed with an 8 mm FWHM. Individual FA quotient images were calculated using the following formula: 100 + 15 × (individual FA–mean)/SD. Regional quotients were then extracted using Johns Hopkins University (JHU) DTI-based white-matter atlases [27] and averaged across regions to produce participant-specific FA-BHQs.

### 2.6. Exclusion Criterion for Outliers

For the data analysis, we defined outliers as follows. First, subjects whose TMT score (TMT Part B – TMT Part A) at baseline was lower than 2 standard deviations from the mean were treated as outliers since this low TMT score implies that the cognition of the subject might be impaired. Then, subjects who had lower than 3 standard deviations from the mean for Stroop or MPI at baseline were also excluded as outliers. Lastly, subjects who have lower than 3 standard deviations from the mean for MRI-derived measures (GM-BHQ, FA-BHQ) at baseline were excluded as outliers since these lower values indicate the poor quality of MR images. In addition, in order to compare subjects as uniformly as possible by reducing the difference in the initial conditions, propensity-score matching was performed with a logistic regression that considered all variables such as age, gender, body mass index, sleeping time, drinking alcohol, and smoking at baseline.

### 2.7. Statistical Analysis

To identify changes due to the intervention over the course of the four-week study period, the symmetrized percent change (SPC) value for each variable was determined and used in the analysis. Assuming that the initial score of the variable *V* is *V*1 and the follow-up score is *V*2, the SPC is obtained as follows: SPC = (*V*2 − *V*1)/(*V*1 + *V*2) × 100. SPC is the rate with respect to the average of the variable *V*. It is more robust than simple percent change because *V*1 can be noisy. First, group differences were investigated for the SPC of each variable between intervention and control groups. Then, a path analysis was performed to analyze how intervention affected the various psychological, cognitive, and MRI-derived brain measures based on the hypothesis that dark chocolate consumption might reduce fatigue, resulting in the improvement of cognitive function and brain structure. All statistical analyses were performed using IBM SPSS Statistics version 26 (IBM Corp., Armonk, NY, USA).

## 3. Results

### 3.1. Demographics

**Figure 1:**
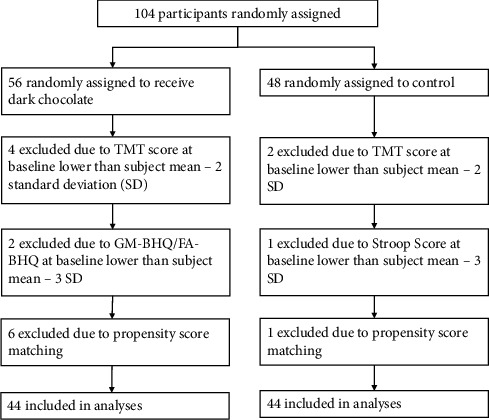
Flow diagram of the trial.

A trial flow diagram for this study is shown in Figure 1. Twelve subjects from the intervention group and four subjects from the control group were excluded from analysis due to being outliers or propensity matching. As a result, 44 participants in the intervention group (24 females, 20 males, ages 40-63, mean age 52.05 ± 7.08 years), and 44 in the control group (20 females, 24 males, ages 40-64, mean age 51.89 ± 6.20 years) were analyzed. As shown in Table 1., there was no significant difference in demographics between the two groups.

### 3.2. Group Differences

The mean consumption of dark chocolate in the intervention group was 4.98 pieces (range: 4.46-5.00) per day, which is equivalent to 631.79 mg (range: 567.14–635.00) of cacao polyphenol. The body mass index change over the four weeks was −0.028 ± 0.536 for the intervention group and +0.043 ± 0.184 for the control group with no significant difference.

**Figure 2:**
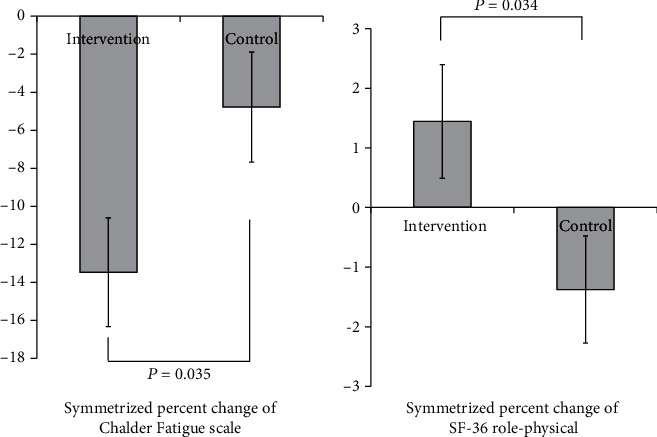
Mean comparison of the physical and mental fatigue score changes and role-physical score changes (Symmetrical Percent Change).

A statistically significant difference was found between the intervention group and the control group in the reduction rate of physical and mental fatigue after the intervention, as is shown in Figure 2 (*p* = 0.035). For reference, this result was almost the same even when the 11-item CFS data were analyzed (*p* = 0.035). However, when the subscales were used, neither physical fatigue nor mental fatigue became significant for either the 14-item or the 11-item versions of the CFS (*p* > 0.05). These results indicate that different people had lower physical and mental fatigue, to varying extents, after the intervention.

Similarly, there was a statistically significant difference in the improvement rate of role-physical, which is role limitations due to physical functioning, measured with the SF-36 between the intervention group and the control group as is shown in Figure 2 (*p* = 0.034). We found no significant differences between the groups for other variables (Table 2).

### 3.3. Path Analysis

**Figure 3:**
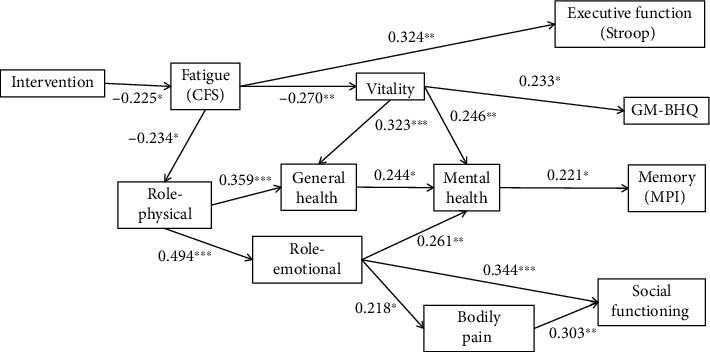
Elucidated association between intervention, fatigue, physical and mental conditions, and brain-derived measures. GFI = 0.950; AGFI = 0.921; NFI = 0.830; CFI = 1.000. *n* = 88; ^∗^*p* < 0.05; ^∗∗^*p* < 0.01; ^∗∗∗^*p* < 0.001. Error term is omitted.

The path analysis revealed that the intervention was negatively associated with physical and mental fatigue worsening and positively associated with improvement of role limitations due to physical functioning (role-physical). Furthermore, these variables were directly or indirectly associated with executive function (Stroop-SPC), memory (MPI-SPC), social functioning, and GM-BHQ through improvement of vitality, mental health, and role limitations due to emotional functioning (role-emotional). These results indicate that the intervention reduced fatigue and improved physical health, and these changes were directly and indirectly associated with gray matter volume, executive function, memory, and social functioning through improvement of various mental conditions (Figure 3).

## 4. Discussion

In this study, we investigated how dark chocolate would affect fatigue, cognitive function, and brain structure. We found that dark chocolate reduced mental and physical fatigue, and directly and indirectly enhanced executive function, memory, social life function, and gray matter volume.

Previous studies have suggested that the cocoa polyphenol included in chocolate might produce antioxidant and anti-inflammatory effects [28], improve cardiovascular health [29], and increase brain-derived neurotrophic factor [30]. These findings led us to consider whether chocolate has a direct effect on the brain, resulting in improved cognitive function. However, our study did not reveal a direct relationship between chocolate consumption and gray matter volume or fractional anisotropy. Neither did we find a direct relationship between intervention and cognitive function. One of the reasons might be the intervention period. Four weeks might not be long enough to cause structural changes of the brain, which would result in cognitive improvement. Instead, we found that chocolate consumption significantly improved the subjective fatigue of the intervention group compared with control, which is in line with previous reports [8, 9]. In addition, subjective reduction of fatigue was positively associated with the Stroop test scores. The Stroop test assesses inhibition, an important factor of executive function. Considering our results, dark chocolate could reduce fatigue, which, in turn, may improve executive function in individuals.

Our study also revealed that fatigue reduction led to the strengthening of vitality, which is related to the deceleration of GM-BHQ reduction. We previously found that the GM-BHQ of individuals who perceived subjective fatigue was lower than those who did not [10]. The results of the current study are consistent with this finding. Furthermore, vitality was related to improvement of mental health as well as MPI, which reflects one's memory function. Fatigue reduction was also associated with an improvement in physical function, which had a positive impact on emotional functioning, relief of bodily pain, and social functioning. Taken together, our results suggest that fatigue may be a mediating factor between dark chocolate consumption and health outcomes. Dark chocolate intake may help reduce the fatigue of individuals, which would lead to the improvement of brain health and various cognitive functions as well as quality of life.

There are some limitations in our study. First, the intervention period was limited to only four weeks. It might take longer to observe brain changes resulting from dark chocolate intake. Second, the sample size was relatively small. Lastly, dietary pattern might affect the results of our study. It is known that Japanese and Mediterranean diets, for example, are already polyphenol-rich [31]. In this respect, we speculate that our intervention might have had a relatively small effect on our subjects because their regular diet may already be polyphenol-rich. In order to eliminate these limitations, more participants from various countries and a longer intervention period might be required.

## Figures and Tables

**(a):** 

	Intervention		Control		*χ* ^2^	*p*
	*n*	%	*n*	%		
Gender						
Male	20	45.5	24	54.5	0.73	0.39
Female	24	54.5	20	45.5		
Sleep duration						
Less than 6 hours	15	34.1	20	45.5	3.97	0.14
6 hours or more and less than 7 hours	21	47.7	12	27.3		
7 hours or more	8	18.2	12	27.3		
Frequency of drinking alcohol						
Do not drink/quit	10	22.7	11	25.0	0.66	0.88
Almost no drinks/1-3 days a month	11	25.0	12	27.3		
1 to 4 days a week	13	29.5	14	31.8		
5 to 7 days a week	10	22.7	7	15.9		
Smoking						
Do not smoke	31	70.5	31	70.5	0.00	1.00
Smoke/previously smoked	13	29.5	13	29.5		

**(b):** 

	Intervention		Control			
	Mean	SD	Mean	SD	*t*	*p*
Demographic variable						
Age	52.05	7.08	51.89	6.20	0.11	0.91
BMI	21.62	3.71	22.61	3.22	-1.33	0.19
Years of education	15.00	2.12	14.89	2.17	0.25	0.80
Psychological condition						
CFS	14.57	7.94	13.61	7.30	0.59	0.56
Physical functioning	55.26	2.00	55.01	2.55	0.51	0.61
Role-physical	50.41	6.50	52.31	7.32	-1.28	0.20
Bodily pain	52.70	8.27	51.15	9.59	0.81	0.42
General health	55.18	7.13	54.30	8.45	0.53	0.60
Vitality	52.54	8.58	50.60	9.36	1.02	0.31
Social functioning	55.01	5.08	55.14	5.07	-0.12	0.90
Role-emotional	51.56	6.68	51.82	7.56	-0.17	0.87
Mental health	56.54	6.75	53.21	10.02	1.83	0.07
Cognitive function						
TMT	32.25	13.90	34.68	20.74	-0.65	0.52
Stroop	4.34	3.73	4.91	4.54	-0.64	0.52
MPI	71.14	9.79	72.21	7.88	-0.57	0.57
MRI-derived measures						
GM-BHQ	99.25	5.06	98.15	5.62	0.96	0.34
FA-BHQ	98.61	3.23	99.08	3.00	-0.71	0.48

**Table 2:** Group comparison of the symmetrical percent change of variables.

	Intervention			Control			*t*	*p*
	Mean	SD	SEM	Mean	SD	SEM		
Psychological condition								
CFS	-13.466	18.947	2.856	-4.781	19.177	2.891	-2.137	0.035^∗^
Physical functioning	0.284	1.214	0.183	0.528	2.175	0.328	-0.651	0.517
Role-physical	1.444	6.333	0.955	-1.376	5.971	0.900	2.149	0.034^∗^
Bodily pain	0.558	8.912	1.344	1.525	12.013	1.811	-0.429	0.669
General health	0.069	5.531	0.834	-0.435	4.121	0.621	0.485	0.629
Vitality	-0.171	4.654	0.702	0.375	5.888	0.888	-0.482	0.631
Social functioning	0.145	4.890	0.737	-0.093	7.228	1.090	0.181	0.857
Role-emotional	1.053	5.177	0.780	-0.516	5.702	0.860	1.351	0.180
Mental health	0.024	3.830	0.577	-0.119	8.763	1.321	0.099	0.921
Cognitive function								
TMT	-3.323	28.646	4.319	-1.193	27.560	4.155	-0.355	0.723
Stroop	10.368	93.386	14.079	29.925	205.960	31.050	-0.574	0.568
MPI	3.086	5.830	0.879	1.770	5.596	0.844	1.080	0.283
MRI-derived measures								
GM-BHQ	-1.927	8.130	1.226	-1.030	5.592	0.843	-0.604	0.548
FA-BHQ	-0.373	4.383	0.661	-0.818	5.267	0.794	0.431	0.667

*n* = 88; ^∗^*p* < 0.05.

## Data Availability

The raw data supporting the conclusions of this manuscript will be made available by the authors, without undue reservation, to any qualified researcher.
